# Frog Swarms: Earthquake Precursors or False Alarms?

**DOI:** 10.3390/ani3040962

**Published:** 2013-10-11

**Authors:** Rachel A. Grant, Hilary Conlan

**Affiliations:** Department of Life Sciences, Anglia Ruskin University, East Road, Cambridge, CB1 1PT, UK

**Keywords:** earthquakes, animal behaviour, amphibians, frogs, toads

## Abstract

**Simple Summary:**

Media reports linking unusual animal behaviour with earthquakes can potentially create false alarms and unnecessary anxiety among people that live in earthquake risk zones. Recently large frog swarms in China and elsewhere have been reported as earthquake precursors in the media. By examining international media reports of frog swarms since 1850 in comparison to earthquake data, it was concluded that frog swarms are naturally occurring dispersal behaviour of juveniles and are not associated with earthquakes. However, the media in seismic risk areas may be more likely to report frog swarms, and more likely to disseminate reports on frog swarms after earthquakes have occurred, leading to an apparent link between frog swarms and earthquakes.

**Abstract:**

In short-term earthquake risk forecasting, the avoidance of false alarms is of utmost importance to preclude the possibility of unnecessary panic among populations in seismic hazard areas. Unusual animal behaviour prior to earthquakes has been reported for millennia but has rarely been scientifically documented. Recently large migrations or unusual behaviour of amphibians have been linked to large earthquakes, and media reports of large frog and toad migrations in areas of high seismic risk such as Greece and China have led to fears of a subsequent large earthquake. However, at certain times of year large migrations are part of the normal behavioural repertoire of amphibians. News reports of “frog swarms” from 1850 to the present day were examined for evidence that this behaviour is a precursor to large earthquakes. It was found that only two of 28 reported frog swarms preceded large earthquakes (Sichuan province, China in 2008 and 2010). All of the reported mass migrations of amphibians occurred in late spring, summer and autumn and appeared to relate to small juvenile anurans (frogs and toads). It was concluded that most reported “frog swarms” are actually normal behaviour, probably caused by juvenile animals migrating away from their breeding pond, after a fruitful reproductive season. As amphibian populations undergo large fluctuations in numbers from year to year, this phenomenon will not occur on a yearly basis but will depend on successful reproduction, which is related to numerous climatic and geophysical factors. Hence, most large swarms of amphibians, particularly those involving very small frogs and occurring in late spring or summer, are not unusual and should not be considered earthquake precursors. In addition, it is likely that reports of several mass migration of small toads prior to the Great Sichuan Earthquake in 2008 were not linked to the subsequent M = 7.9 event (some occurred at a great distance from the epicentre), and were probably co-incidence. Statistical analysis of the data indicated frog swarms are unlikely to be connected with earthquakes. Reports of unusual behaviour giving rise to earthquake fears should be interpreted with caution, and consultation with experts in the field of earthquake biology is advised.

## 1. Introduction

Anomalous behaviour in both terrestrial and aquatic animals prior to large earthquakes has been reported widely in both the scientific and the popular literature [1,2]. Although most reports of unsual behaviour prior to earthquakes are anecdotal [3,4] recent evidence on physical and chemical processes occurring beneath the earth’s crust in the earthquake preparation zone has enabled a possible causal mechanism for diverse precursory pheomena inclusing unusual behaviour to be proposed [5,6].

Amphibians and earthquakes have long been linked in mythology in earthquake prone areas of the world [7], along with certain other animals such as catfish. This has been the case for many years in many parts of Asia, for example Mongolia, where the earth was said to rest on the back of a giant frog, which caused earthquakes by moving various body parts [7]. It has been suggested [7] that particular animals became linked with earthquakes because they exhibit unusual behaviour prior to seismic activity although there is no firm evidence for this. Anecdotally, snakes and frogs have been reported to come out of hibernation prior to earthquakes [2,8].

Recently, unusual behaviour of anuran amphibians (frogs and toads) has been linked to subsequent large earthquakes. In Sichuan province, China, thousands (some reports say hundreds of thousands) of small toads were seen crossing a road, two days before the catastrophic Great Sichuan Earthquake (M = 7.9) on 12 May 2008. This occurrence, although anecdotal, has crept into the scientific literature; as well as being reported on various Chinese news sites. For example, the phenomenon was reported in the UK’s Daily Telegraph newspaper [9] and was also mentioned in the journal Nature [10] as well as several peer reviewed papers [6,11]. However, until now, the occurrence has never been investigated systematically. Other unusual behaviour of amphibians has been linked to seismic activity; in April 2009, common toads (*Bufo bufo*) abandoned spawning and left their breeding site five days before the L’Aquila, Italy (M = 6.3) earthquake on 6 April 2009 and only returned after the quake had occurred [12].

Possibly because of these two incidents, recent large amphibian migrations have triggered media speculation that they may be precursors to significant earthquakes. In particular, a mass migration of frogs in Thessaloniki, Greece, in May 2010 [13] gave rise to numerous media reports suggesting a large earthquake was imminent in Greece or neighbouring Turkey, but no earthquake occurred. Similarly, frog swarms occurring in Moratuwa, Sri Lanka, in May 2010 [14] also gave rise to earthquake fears, while in 2011 and 2012 several frog swarms have caused unfounded anxiety in Wuhan and Nanjing, China [15]. Avoidance of false alarms is of utmost importance in short term earthquake risk forecasting to preclude panic among populations living in seismic risk areas.

Many amphibians, particularly in temperate regions, breed in spring and their larvae metamorphose into juvenile frogs and toads in summer [16]. In many cases, this metamorphosis and dispersal away from their natal pond is synchronised, leading to large aggregations of tiny frogs or toads. Synchrony of metamorphosis and post-metamorphic aggregations are probably a defence against predation; large groups of animals moving together minimises predation on the individual [17,18]. Hence, after a successful breeding season, it is not uncommon to see large numbers of tiny juvenile toads or frogs migrating from the breeding site and dispersing to terrestrial habitats [19]. Where these coincide with roads or residential areas, they may find their way to media reports as “frog swarms”. In the temperate Northern hemisphere these aggregations normally occur in late spring or summer, shortly after metamorphosis has occurred. In dry climates a further migration may be seen in early autumn (fall) after aestivation. Therefore, it is possible that frog swarms are not linked to large earthquakes, but are part of the normal migratory behaviour of juvenile anuran amphibians. If this is the case, they would be expected to occur primarily in summer, and to consist of very small, uniformly sized amphibians.

For an amphibian migration to be newsworthy, it must by definition be large enough to be seen as unusual. Most frog migrations are probably unnoticed, occurring in rural areas away from roads and residential areas. Frog swarms do not occur every year as they depend on numerous climatic and other factors [20] and the size of many amphibian populations is highly variable from year to year. 

In this study, news reports of large and “unusual“ frog and toad migrations were searched for and earthquake data examined in the area to evaluate what proportion of “frog swarms” reported in the media were followed by a significant level of seismic activity. Temporal spacing of frog swarms and the size of the animals were noted to determine whether they were juvenile post-metamorphic aggregations.

## 2. Methods

As most previously reported unusual animal behaviour before earthquakes occurs within 100 km of the earthquake’s epicentre [21], only earthquakes occurring within this radius were considered in relation to frog swarms. Most unusual behaviour prior to earthquakes is reported to occur a day or two before the earthquake, but may be seen up to a month or two prior to the event [21]. 

Therefore earthquakes up to one month after the frog or toad swarm occurred were included. The majority of anomalous animal behaviour is reported to occur prior to earthquakes above M = 4.5 [21] so earthquakes above M = 4.5 were considered.

News reports of large amphibian migrations between 1850 and 2010 (inclusive) were searched for, using the search terms “frog swarm”, “toad swarm”, “unusual behaviour toad”, “unusual behaviour frog”, “frog plague”, “toad plague”, “mass migration frog”, “mass migration toad” “hundreds/thousands of frogs/toads”. Searches were carried out both on the standard google site (www.google.com) and Google News Archive, adapting the methods for systematic searches of online news stories given by Habel *et al.* [22] (news.google.com/archivesearch), but limited to the English language. Reports were entered into a database and then categorised depending on the reliability of the reports. Incidents where photographic or video evidence was present, or the occurrence was reported in a newspaper, were classed as reliable. Internet blogs without photographic or video evidence were classed as potentially unreliable and were excluded from the analysis. Hence, many reports of frog swarms, which occurred on blog sites, were excluded. Data on significant (M > 4.5) earthquakes within 100 km radius of the frog swarm and up to one month later, were obtained from the United States Geological Survey (USGS) earthquake search database, using the circular search facility with a radius of 100 km from the position of the frog swarm. The USGS earthquake search facility can be accessed at the following link: http://earthquake.usgs.gov/earthquakes/eqarchives/epic/epic_circ.php. The “USGS/NEIC (PDE) 1973 onwards” database was used. For frog swarms before 1973 a combination of searches were carried out including state by state searches for the USA, available from the USGS, and their historical earthquakes database which can be accessed at http://earthquake.usgs.gov/earthquakes/states/historical.php. 

Using the description and/or video or photographic evidence available, frog and toad “swarms” were classified as either juvenile or adult migrations. This classification was based mainly on size. Amphibians described as “tiny”, “minute”, “dime-sized”, “half grown”, ”froglets”, “toadlets” “half-inch” “thumbnail-sized” and “baby-frogs” in news reports were classified as juveniles. Where photographs or video showed that the animals were very small (2 cm long or less) and very uniform in size, these were also classed as juveniles. 

### Statistical Analysis

We used an application of medical statistics, *i.e.*, a case-control study [23] to test the hypothesis that a reported frog swarm is connected with a higher probability of earthquake occurrence. In medical statistics, case control studies are used to identify risk factors for diseases that are rare. In such cases, it is not feasible to choose random sets of people who are and are not exposed to a risk factor, because the follow-up period and/or sample size would have to be extremely large. Instead, one identifies cases where the disease occurs, finds a set of otherwise comparable cases, and compares the number that were exposed to the putative risk factor to test for an association between the two. The same logic applies when testing for an association between any condition and rare outcome, regardless of whether the outcome is a disease. Hence we used the case control study to look at associations between earthquakes and frog swarms. We used our collected data on the days on which frog swarms occurred, and we picked the same number of random dates which formed the control group. We then looked at whether an earthquake did or did not occur within 1 month and 100 km of the frog swarm.

We also used a Chi-Squared test to test the hypothesis that frog swarms occurred more frequently on the West (seismically active) and East (low seismic activity) of the USA, and a Chi squared test to test for differences in the month in which frog swarms occurred. Small Stata 11 was used for the case-control study and Minitab 14 for the Chi-squared test. 

## 3. Results

There were 28 frog swarms included in the analysis.

### 3.1. Geographical Distribution of Frog Swarms

**Figure 1:**
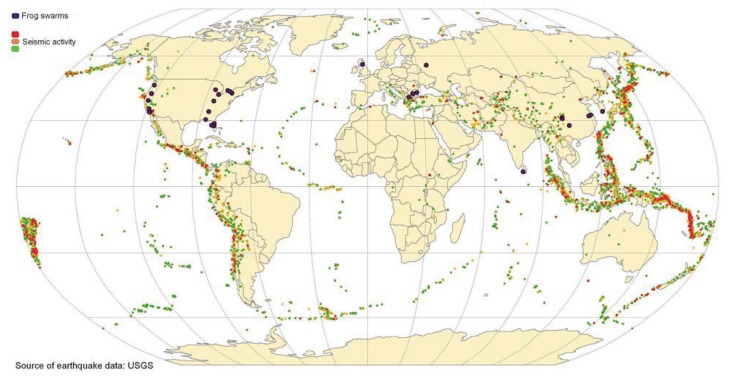
Geographical distribution of frog swarms in relation to seismic risk zones.

Frog swarms occurred in both seismically active and areas of low seismicity (Figure 1). Many of the frog swarms were reported in the USA, probably because of the bias inherent in using Google News Archive, and the English language. Fortuitously, the East–West coast dichotomy proves useful for the purposes of this study; the West coast being highly seismically active and the East coast generally being inactive (Figure 1). Ten frog swarms were reported on the East coast and only five on the West coast, which is not a significant difference, thereby supporting the null hypothesis, that frog swarms are not associated with seismic risk zones (Chi-squared = 1.67, N = 15, *p* = 0.2). 

### 3.2. Temporal Distribution of Frog Swarms

**Figure 2:**
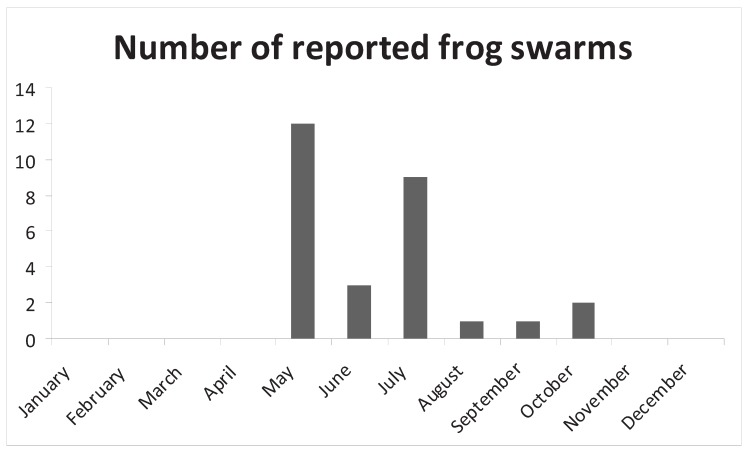
Temporal distribution of frog swarms (from 1850–2010).

The majority of frog swarms occurred in May, June and July, with smaller numbers occurring in August, September and October (Figure 2). No frog swarms occurred from November to April inclusive. In order to analyse the data by goodness of fit tests, they were grouped into seasons (winter = December, January and February; spring = March, April and May; summer = June, July, August; autumn (fall) = September, October, November). There were significant differences between the numbers of frog swarms in each season (winter 0; spring 12; summer 13; autumn 3; chi squared test n = 28, df = 3, Chi-sq = 18, *p* < 0.001) which is to be expected as amphibians hibernate in winter. If the frog swarms related to adult frogs, it would be expected that more would be seen in spring (March and April) which is the breeding season (in the temperate northern hemisphere [11]), however most frog swarms occurred in the months of May, June and July which is when metamorphs of spring breeding amphibians would be expected to disperse [11]. 

### 3.3. Frog Swarms Followed by a Significant (M > 4.5) Earthquake within 100 km Radius

**Figure 3:**
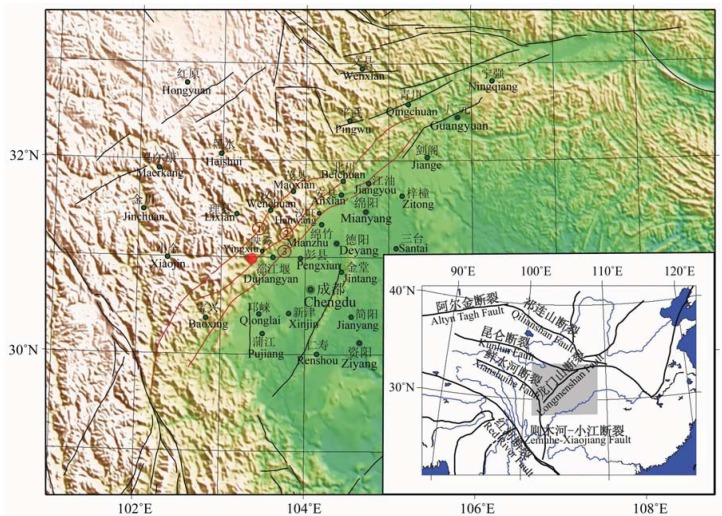
The Longmenshan thrust fault. From Wang *et al*. (2008) [26].

Out of the 28 frog swarms, two of them were followed within a month by an earthquake of M > 4.5 within 100 km of the swarm. A frog swarm occurred on the 9 May 2008, in Mianzhu City, which preceded the earthquake Great Sichuan Earthquake of 12 May 2008 and fits the criteria for inclusion in the analysis (Table 1). However it should be noted that a week after the Great Sichuan Earthquake, a further toad swarm was seen (Table 1), on 19 May 2008, giving rise to speculation that another earthquake was imminent. However no earthquake of significant magnitude occurred. In 2010 a frog swarm on 5 May in Sichuan Province, China preceded a moderate earthquake of M = 5 on 25 May, however another frog swarm on 11 May 2010 in Nanjing did not precede any seismic activity (Table 1). A large migration of small toads occurring on 10 May 2008, in Taizhou, Jinangsu, which preceded the Great Sichuan Earthquake of (Figure 3), news of which was widely disseminated, in fact occurred more than 1,500 km away from the epicentre so was excluded from the analysis. 

The case control study showed no statistically significant association between a reported frog swarm and a future earthquake (McNemar’s Exact test b = 2, c = 0, *p* = 0.5). 

### 3.4. Detail on the Great Sichuan Earthquake (M = 7.9; 12 May 2008)

**Figure 4:**
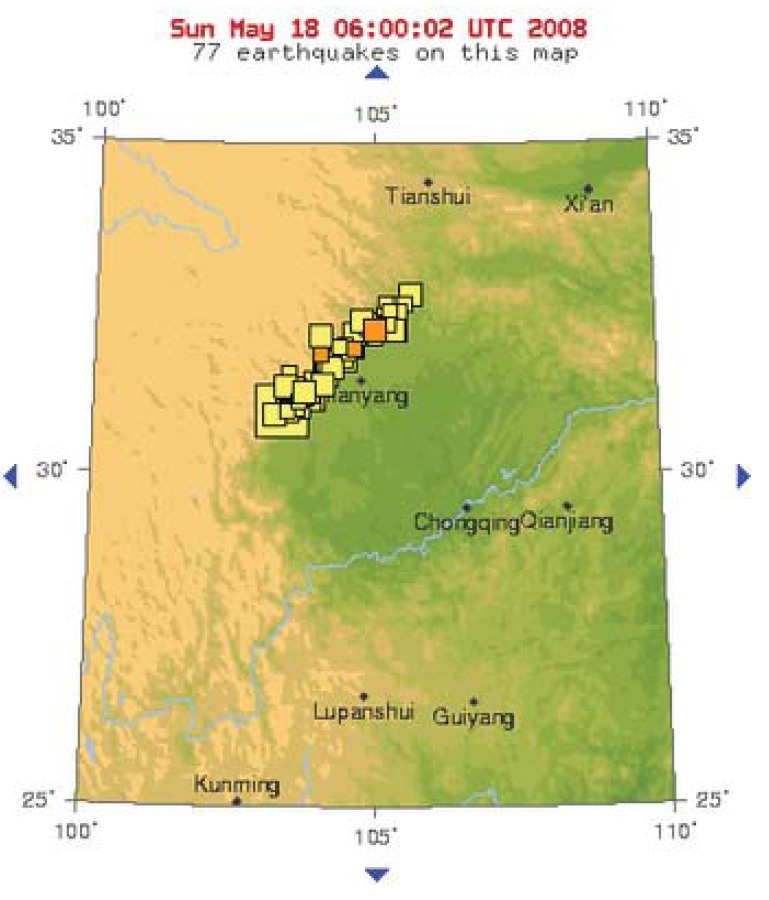
Map showing epicentre and earthquakes relating to the great Sichuan Event of 12 May 2008 (source: USGS).

The Great Sichuan Earthquake (also called Wenchuan earthquake) occurred at the NE-SW striking Longmenshan thrust fault (Figure 3) in Southwest China on 12 May 2008 at (06:28UT, 14:28LT) with magnitude (Mw =7.9) with a depth of 19 km [24,25]. The epicentre (31.0°N, 103.4°E; (Figure 3 and Figure 4)) of the earthquake was situated approx 80 km WNW of Chengdu, the province’s capital city [20], causing massive destruction and loss of life.

**Table 1:** Frog swarms from 1850 to 2010.

Location	Date	Latitude	Longitude	EQ
Pleasant Township, IN, USA	19 July 1892	38.8828376	−85.0987487	
Ithaca, NY, USA	07 July 1901	42.443914	−76.5018807	
Churchville, NY, USA	22 May 1903	43.1042277	−77.8844543	
Kalamath Falls, OR, USA	24 July 1913	42.224867	−121.7806704	
Ludington, MI, USA	23 July 1929	43.9552825	−86.4525830	
Alabama City, AL, USA	07 August 1949	34.0223194	−86.0455285	
Kalama, WA, USA	03 October 1951	46.0084477	−122.84455	
Old Badly, Korea	27 July 1952	38.228611	127.0036110	
Gardena, CA, USA	12 May 1958	33.8883487	−118.3089623	
Anaheim, CA, USA	04 July 1980	33.8352932	−117.9145099	
Seminole, FL, USA	22 September 1981	27.8397466	−82.7912	
Toronto, Ontario, Canada	27 July 1994	43.653226	−79.3831843	
Mozhaysk, Russia	23 June 1995	55.5155707	36.0433416	
Fort Walton Beach, FL, USA	17 October 2002	30.4200707	−86.61703	
**Mianzhu city, Sichuan, China**	**09 May 2008**	**31.338077**	**104.220749**	**Great Sichuan Earthquake M = 8 on 12 May 2008**
Taizhou, Jiangsu, China	10 May 2008	32.455778	119.923115	
Bakersfield, CA, USA	15 May 2008	35.3732921	−119.087124	
Zunyi, Guizhou, China	19 May 2008	27.725654	106.927388	
Blissfield, MI, USA	27 June 2008	41.7640537	−83.7492083	
Edinburgh, UK	04 July 2008	55.953252	3.1882670	
**Chengdu, Sichuan, China **	**05 May 2010**	**30.658601**	**104.064855**	**Earthquake M = 5 on 25 May 2010**
Nanjing, Jiangsu, China	11 May 2010	32.060255	118.796877	
Moratuwa, Sri Lanka	13 May 2010	6.796396	79.877823	
Thessaloniki, Greece	26 May 2010	40.63935	22.944607	
Lake Apopka, FL, USA	26 May 2010	28.6239487	−81.6254283	
Black Sea, Bulgaria	30 May 2010	42.6603497	27.7179353	
Chirpan, Kremena Daneva, Bulgaria	18 June 2010	42.1995609	25.3251252	
Marysville, CA, USA	02 July 2010	39.1534778	−121.5859025	

The general tectonics of the area relate to the movement of the Indian plate relative to the Eurasian plate, causing eastward deformation of the Tibetan plateau, halted by the Sichuan basin [27]. The largest intra-continental earthquake in western China ever recorded by instruments produced a 300 km rupture belt which is characterised by an oblique-reverse focal mechanism (thrust uplift and right-lateral strike slip) with maximum vertical dislocation of 6.2 m and horizontal dislocation of 4.9 m. 

Several phenomena which can be seen to be precursory occurred before the Wenchuan earthquake. Anomalous measurements of vertical total electron content (VTEC) occurred with anomalously high values on 3 and 9 May, while the anomalous decreases appeared on 29 April and 6 May [28]. Hsiao *et al.* [29] also noted anomalous changes in electron density detected by satellite within 5 days of the earthquake. 

**Figure 5:**
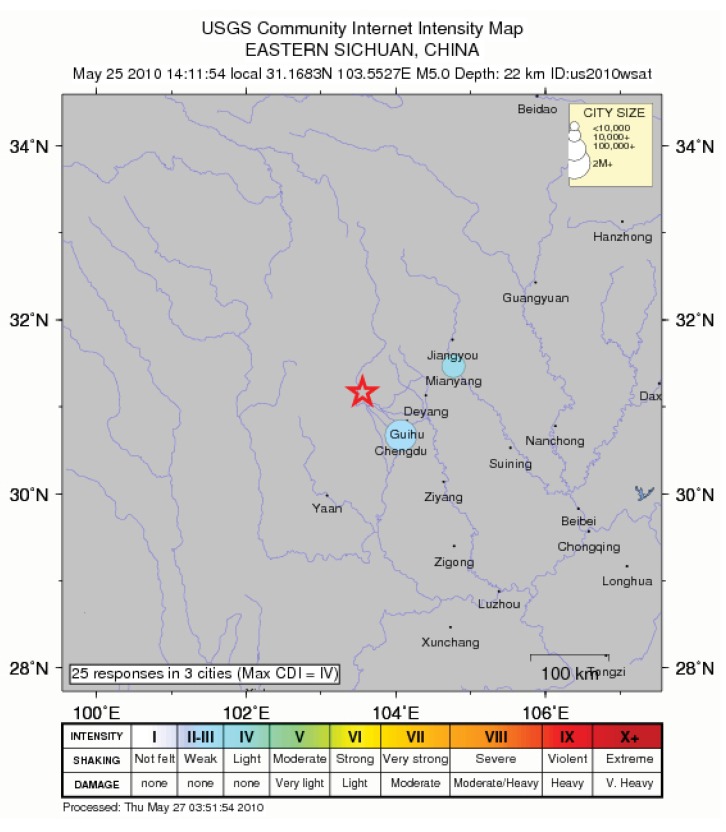
The epicentre of the Chengdu earthquake of 5 May 2010 (M = 5) (source: USGS).

The Chengdu earthquake of 5 May 2010 (M = 5) may have been an aftershock of the Great Sichuan event, occurring at a very similar latitude and longitude (Figure 5).

### 3.5. Number of Frog Swarms which Comprised Juvenile Anurans in Post-Metamorphic Aggregations

**Figure 6:**
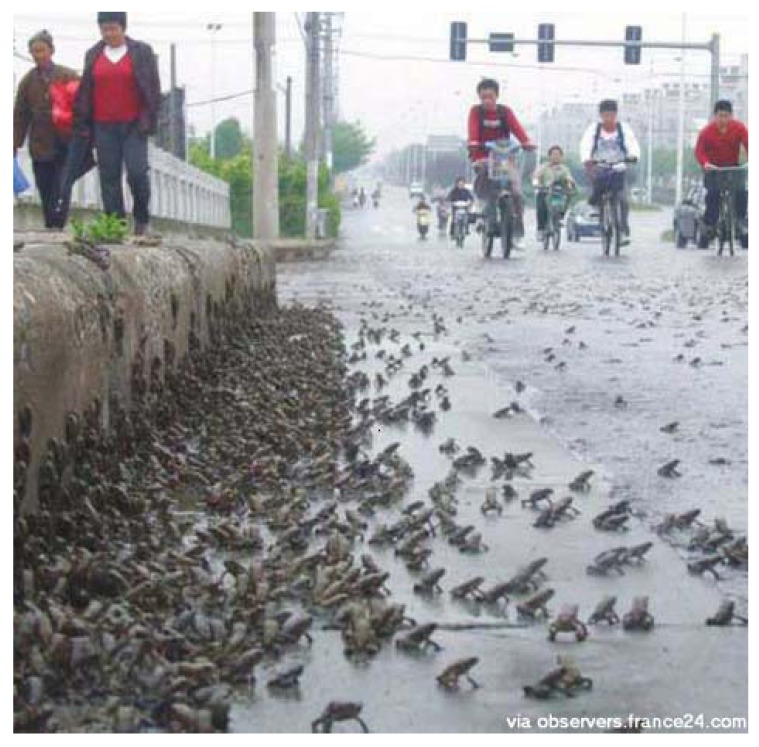
A migration of toads crossing a street in Taizhou, Jiangsu province, China on 10 May 2008, two days before the great Sichuan Earthquake, but more than 1,500 km from the epicentre. Notice the small, uniform size of the anurans (source: France 24 International news site).

**Figure 7:**
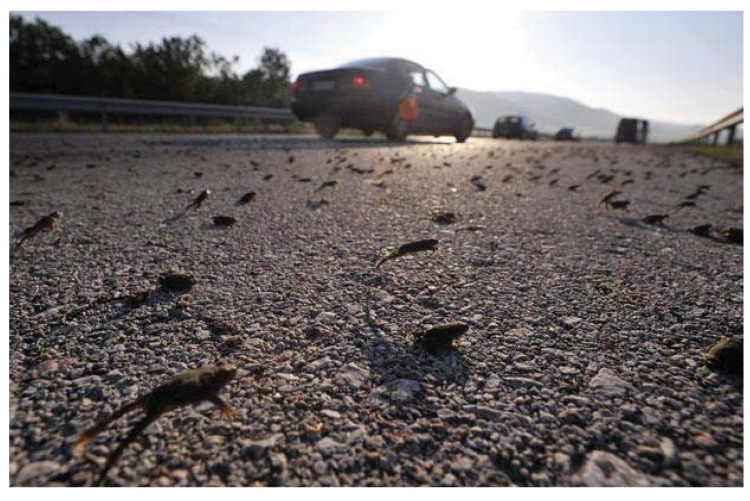
Frogs crossing a Greek highway on 27 May 2010. Again, the small and uniform size suggests a juvenile migration (source: Daily Telegraph Newspaper, UK).

**Figure 8:**
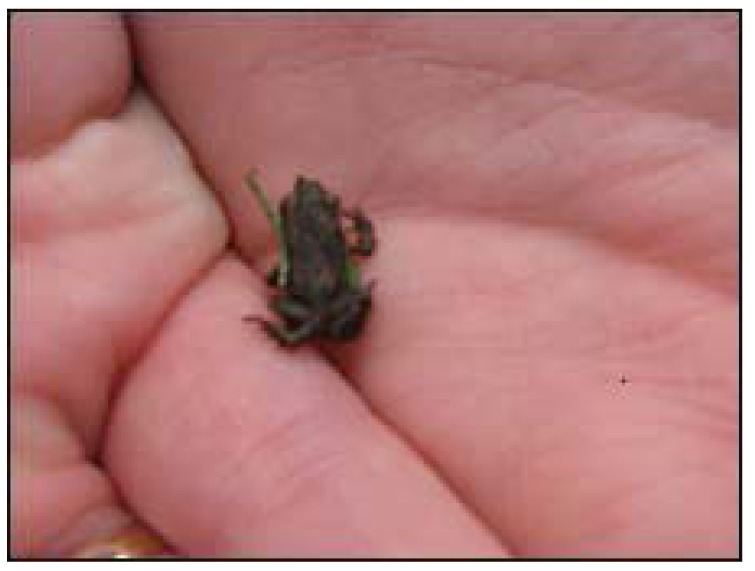
Thousands of tiny toads stopped traffic in Edinburgh, UK (an area of generally low seismic activity) on 4 July 2008 (source: BBC News www.bbc.co.uk/news).

Using the criteria set out in the methods section, all of the frog swarms were classified as juvenile migrations. Figure 6, Figure 7 and Figure 8 show the small uniform size of the anurans in typical frog swarms. 

## 4. Discussion

All of the reports of frog swarms that were reported appeared to relate to very small juvenile anurans and the majority of these occurred in early summer (May, June and July). Only two of these were associated with earthquakes. It is likely that this was a co-incidence as the time of year (May) and the size of the frogs or toads indicate a juvenile aggregation. The statistical tests carried out supported the null hypothesis; i.e. there is no link between frog swarms and earthquakes. 

There is likely to be a bias in reporting frog swarms in seismic risk zones. The media in earthquake risk areas are inherently interested in frog swarms because of mythology which links them to earthquakes. So our data are biased towards those frog swarms occurring in seismic risk areas being more likely to be reported and widely disseminated. For example, the frog swarm occurring prior to the Great Sichuan Earthquake was reported in Chinese news sites at the time it occurred, but was widely disseminated on the internet in English only after the earthquake, further biasing the data. Because of the reporting biases discussed it is not always possible to disentangle real effects from the effects of reporting bias. Therefore, media reports of animal behaviour abnormalities in general may not be useful in forecasting earthquake likelihood.

**Figure 9:**
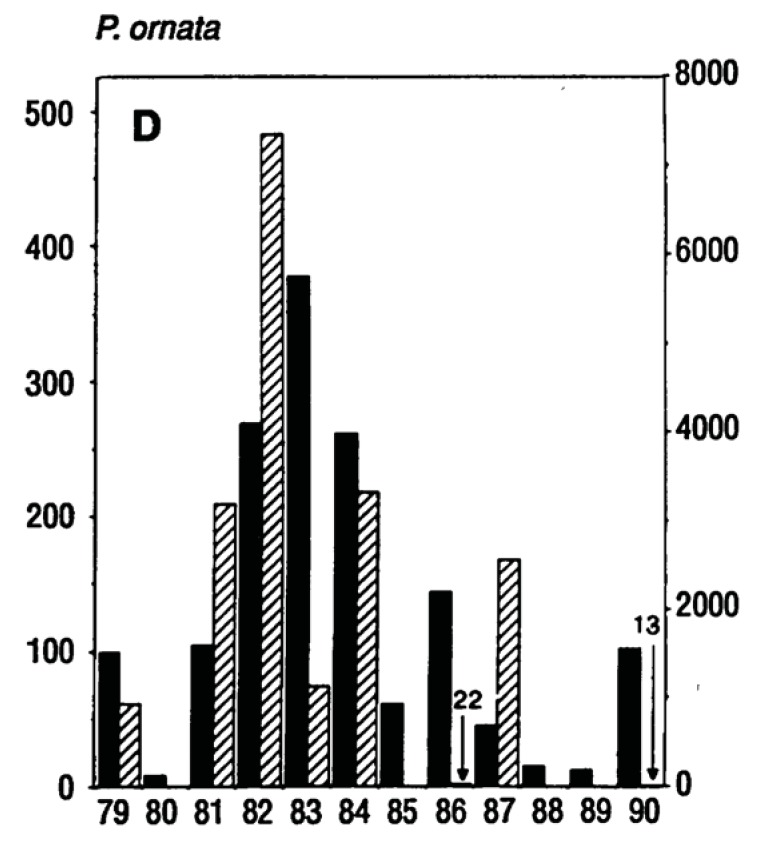
Fluctuation of populations of *Pseudacris ornata* (a spring breeding anuran) from 1979 to 1990. Solid bars indicate the numbers of breeding females and hatched bars indicate metamorphosing juveniles. The right hand axis shows juvenile numbers. From Pechmann *et al.* [30].

Amphibian population numbers depend on successful reproduction which depends on a complex array of environmental correlates. In the wood frog (*Rana sylvatica*), a typical spring breeding anuran amphibian, adult numbers can vary by a factor of 10 from year to year and juveniles by a factor of 100 [20]. In other species the fluctuations are even more dramatic; Pechmann *et al.* [30] reported population fluctuations in the *Pseudacris ornata* (another spring breeder) from 0 juveniles (in 1980) to more than 7,000 (in 1982) and back to 0 in 1985 (Figure 9). 

Thus it can easily be seen juvenile frog swarms do not occur every year. The production of a large number of juveniles depends on multiple environmental factors including the correct amount of rainfall—adequate moisture is needed for reproduction [20]. Long periods of dry weather will reduce the numbers of juveniles recruited [20], as will low food availability, an increase in the predator population, ephemeral pond drying and water depth [31], parasitism and disease [20]. A minimum threshold temperature is required for physiological functioning and reproduction and in very cold springs breeding can be delayed [32]. Reading and Clarke (1995) [33] found that fecundity in the common toad (*Bufo bufo*) was affected by body condition which in turn depended on numerous factors including rainfall during the summer before breeding and the average temperature of the month before spawning occurred. 

The work of Grant and Halliday [10] and Grant *et al.* [6] detailed unusual behaviour in anuran amphibians (specifically, *Bufo bufo*, the common toad) prior to the M = 6.3 L’Aquila earthquake on 6 April 2009. Toads had been studied at the site for the four years preceding the earthquake, and had showed normal activity, mating and spawning continuously for a period of 3–6 weeks each spring and not leaving the site until spawning was completed. Prior to the earthquake, however, and coinciding with disturbances in the ionosphere detected by radio sounding methods, toads disappeared from the breeding site, only returning when the earthquake was over. This behaviour could be deemed “unusual” as normal behaviour over the preceding four years had been recorded. 

This behaviour differs in several respects from the “frog swarms” reported in the media. The behaviour noted by Grant and Halliday) [10] involved an unusual sharp decrease in numbers at a time of year when numbers could be expected to be high. The data could be compared with that of the previous 4 years and backed up by literature on what is usual for spawning common toads; this could be deemed to be unusual. Frog swarms however, are somewhat opposite in character to the observations of Grant and Halliday [10]. Grant and Halliday noted a reduction in amphibian numbers, but frog swarms are examples of very many amphibians moving in a group large enough to attract media attention. Therefore the two types of observations have little in common, apart from that they concern amphibians. Due to mythology, anecdotal reports of frogs coming out of hibernation before earthquake and (occasionally) misinterpretation of Grant and Halliday’s (2009) [10] observations, media reports portray frog swarms as unusual behaviour, leading to public anxiety. What this paper attempts to demonstrate is that large numbers of migrating small frogs are normal behaviour and therefore cannot be compared at all with the observations of Grant and Halliday (2009) [10], who recorded *unusual* behaviour. Small frogs moving in large groups in spring and summer are probably juvenile migrations. What would be unusual would be to see this phenomenon in winter, when anurans hibernate due to temperatures being below the threshold for physiological activity. However, none of the reported frog swarms occurred in winter.

While it is likely that electromagnetic, geophysical and geochemical changes prior to large earthquakes do affect many animals, giving rise to abnormal behaviour [6], animal behaviour needs to be evaluated by comparing the behaviour with that which is normal for that species or group, before a conclusion can be reached. Post-metamorphic aggregations of juvenile amphibians in summer are not unusual behaviour. 

The number of frog swarms detected by our search criteria is relatively low, perhaps lower than could be expected for a global search. Large areas of the world appear to have no reported frog swarms in the study period. The possible reasons for this are as follows:

(1) As previously discussed, only reports in the English language were used. The areas of highest seismic risk are generally found in non-English speaking countries, as are highest biodiversity of amphibians.
(2) Many of the reports occurred on internet blog sites and did not provide photo or video evidence, so were excluded from the analysis as being potentially unreliable
(3) Amphibian populations undergo enormous fluctuations (several orders of magnitude) in population size from year to year as mentioned in the introduction due to climatic and other variables, meaning frog swarms will not occur every year. Evidence for this can be seen in China where frog swarms occurred in 2008 and 2010 but not 2009.
(4) Many amphibians in subtropical and tropical locations breed all year round as the climate is favourable for egg and larval development and food is abundant [34,35]. Therefore only amphibians in temperate climates are spring breeders where populations metamorphose in synchrony over short periods of time [36,37]. Hence most frog swarms will be expected to occur in temperate regions.
(5) Frog swarms may not always be reported when they occur away from roads and other urban centres, which is likely to relate to most frog swarms, as amphibian breeding sites are often away from areas of human disturbance.

## 5. Conclusions

The data presented indicate that frog swarms are not unusual behaviour for amphibians and are consequently not earthquake precursors. Furthermore, all of the frog swarms reported in this study were probably juvenile migrations of newly-metamorphosed anurans. Large amphibian migrations, being normal behaviour, are unlikely to be useful in forecasting earthquake risk, although genuinely unusual behaviour of amphibians has been shown to be associated with earthquakes [16]. Reports of suspected unusual animal behaviour in seismic hazard locations should be interpreted with caution, and consultation with experts in the field of earthquake biology is advised to avoid false alarms. When using media and anecdotal reports, awareness of possible reporting and recollection bias should be maintained. 
